# Patent Medicines in Drug Stores

**Published:** 1879-04

**Authors:** 

**Affiliations:** Goodrich, Mich.


Patent Medicines in Drug Stores.—As the question of the qualification of the doctor to practice medicine is now being agitated, would it not be well to bring another question to the front, which every doctor of medicine is directly interested in, and on which it wants only a concert of action to be of great benefit to the profession and the people—I refer now to the patent nostrums which fill the shelves of every drug shop in the land. The relation of physician and druggist is of vital importance when properly regulated; but under the existing state of affairs it is decidedly against the physician and in favor of the drug man—and this thing ought not to be so. Retail druggists work for money, and they, as a class, do not care how they get it—would as soon sell patent nostrums as fill prescriptions—and they will recommend the stuff right in the face and eyes of a physician giving them the patronage of his prescriptions. I would recommend and urge every physician to withdraw his patronage from all such dispensers of stuff, and compel them to empty their shelves of everything of a nostrum kind, and give the physician his honest due for labor performed. Who will second the motion? We have the remedy in our own hands, and let us use it unless they come to time. DR. HOWELL.

*Goodrich, Mich., March* 15, 1879.

[*Mich. Med. News.*

